# A Case of Severe Mpox Complicated with *Streptococcus pyogenes* Sepsis in a Patient with HIV Infection

**DOI:** 10.3390/pathogens12091073

**Published:** 2023-08-23

**Authors:** Silvia Di Bari, Annalisa Mondi, Carmela Pinnetti, Valentina Mazzotta, Fabrizio Carletti, Giulia Matusali, Donatella Vincenti, Roberta Gagliardini, Raffaele Santoro, Carla Fontana, Fabrizio Maggi, Enrico Girardi, Francesco Vaia, Andrea Antinori

**Affiliations:** National Institute for Infectious Diseases ‘Lazzaro Spallanzani’ (IRCCS), 00149 Rome, Italy

**Keywords:** monkeypox infection, HIV, *Streptococcus pyogenes*, multi-mucosal involvement

## Abstract

Since May 2022, a global outbreak of human Mpox has rapidly spread in non-endemic countries. We report a case of a 34-year-old man admitted to hospital for a six-day history of fever associated with vesiculo-pustular rash involving the face, limbs, trunk and perianal region, lymphadenopathy and severe proctitis and pharyngitis. He was HIV-positive and virologically suppressed by stable antiretroviral therapy. On admission, Mpox virus-specific RT-PCR was positive from multiple samples. Additionally, blood cultures yielded *Streptococcus pyogenes*, prompting a 14-day-course of penicillin G and clindamycin. Due to the worsening of proctitis along with right ocular mucosa involvement, tecovirimat treatment was started with a rapid improvement in both skin and mucosal involvement. The patient was discharged after 21 days of hospitalization and the complete clinical resolution occurred 38 days after symptom onset. This is a case of Mpox with extensive multi-mucosal (ocular, pharyngeal and rectal) and cutaneous extension and *S. pyogenes* bacteraemia probably related to bacterial translocation from the skin or oral cavity that was eased by Mpox lesions/inflammation. The HIVinfection, although well controlled by antiretroviral therapy, could have played a role in the severe course of Mpox, suggesting the importance of a prompt antiviral treatment in HIV-positive patients.

## 1. Introduction

Mpox (formerly known as monkeypox) is a zoonotic disease caused by Mpoxvirus (MPXV), a double-stranded DNA virus belonging to the *Orthopoxvirus* genus of the Paxviridae family [1]. Two genetic clades of MPXV have been described: the Central African clade, responsible for a more severe illness, and the West African clade [2]. Mpox has historically been considered endemic in Central and West Africa [3],with sporadic cases or outbreaks outside endemic regions associated with returning travellers [4,5,6] or importation of infected animals [7].However, since May 2022, a global outbreak of Mpox has begun and spread rapidly in non-endemic countries, affecting over 88,600 people to date [8]. The 2022 outbreak has been driven primarily by human-to-human transmission and mostly affected men who have sex with men (MSM) [9]. The temporal association with sexual activities, the peculiar localization of Mpox lesions and the detection of MPXV-DNA and infectious virus in semen, has strongly suggested an important role for sexual transmission [9,10,11].

Classical clinical presentation of Mpox is characterized by initial, non-specific, systemic symptoms including fever, asthenia and lymphadenopathy, followed by asynchronous maculopapular and vesicular rashes with centrifugal distribution [12]. Peculiar clinical features have been described during the 2022 outbreak including the predominant localization of lesions in genital, ano-rectal and oro-pharyngeal regions and the frequent mucosal involvement with proctitis and pharyngitis [9,12,13].

During the current outbreak, Mpox has been generally described as a self-limiting disease with a low fatality rate. However, severe complications, including lung and ocular involvement, encephalitis and secondary bacterial infection, have been reported, especially in subjects with underlying conditions such as immunodeficiency [9,12,13], including those with HIV infections who have been disproportionately affected during this outbreak, accounting for 38–50% of Mpoxcases [9,13,14].

Secondary bacterial infections have been reported as complications of Mpox in several case series and mostly described as mild and localized [9,13,14,15]. Although the diagnosis of this complication is challenging due to the overlapof clinical manifestations between the two conditions, its prompt identification and management is crucial to set the correct therapy and avoid excessive use of unnecessary antibiotic treatments, as already reported in previous studies [13,15].Here, we describe a clinical case of Mpox with severe multi-mucosal involvement and systemic bacterial superinfection by *S. pyogenes* causing bacteraemia in a HIV-positive patient.

## 2. Case Description

In 2022, a 34-year-old male was admitted to the National Institute for Infectious Diseases ‘Lazzaro Spallanzani’ for a 6-day history of high fever followed by the appearance of an asynchronous vesiculo-pustular rash involving the face, trunk and perianal region associated with latero-cervical and inguinal lymphadenopathy, sore-throat and severe rectal pain with mucous discharge and bleeding.

The patient had a past medical history of well-controlled HIV infection (stage A2) since 2012 and was on stable antiretroviral therapy (ART) with dolutegravir and lamivudine and previous primary syphilis had been treated with penicillin some years before the hospitalization. Of note, he did not report a history of smallpox vaccination. The patient self-identifies as MSM with a history of unprotected intercourse with multiple partners, the last of whom was within 3 weeks from symptom onset. 

On hospital admission, he was pyretic and complained of severe rectal pain. The initial medical examination confirmed multiple vesicular lesions on the face (n = 3), trunk (n = 5) and perianal region (n = 10), pharyngeal hyperaemia with two ulcers on the right tonsil and painful inguinal and latero-cervical lymphadenopathy. His lymphocyte CD4 count was 652 cell/mm^3^ and his HIV viral load was undetectable. Screening for sexually transmitted diseases (STDs) was negative for concomitant infections. Swabs for MPXV from multiple skin and perianal lesions, oropharyngeal swab (OPS) and blood cultures were collected. Approximately 48 h after hospitalization, the isolation of *S. pyogenes* from initial blood cultures was communicated by the laboratory. According to the antibiogram showing thesensitivity of the isolate to all the tested antibiotics except for quinolones and tetracyclines, intravenous antibiotic treatment with clindamycin (600 mg every 8 h) and penicillin G (4 MUI every 4 h) was immediately started and continued for 14 days, with a rapid defervescence. A trans-thoracic echocardiogram was negative for endocarditis and the 10 day follow-up blood cultures were negative. Positive results of MPXVpolymerase chain reaction (PCR) from both OPS and perianal area and cutaneous lesions were available 72 h after hospitalization. Specific Mpox treatment was initially delayed because of the hospital and national lack of tecovirimat. Since hospitalization day 2, a progressive worsening of clinical conditions was observed with the appearance of new multiple vesiculo-pustular lesions on the limbs including palms and soles, scalp and penis and a significant extension of the rash on the previously affected areas, in particular, on the perianal region and oropharyngeal mucosa. Concurrently, an exacerbation of both the proctitis with an increase in abdominal/rectal pain and rectal bleeding and pharyngotonsillitis with oral intake limitation occurred. Furthermore, three days after hospital admission, involvement of the right ocular mucosa was observed, with the appearance of a painful vesicular lesion on the inferior eyelid, near the inferior lacrimal punctum, which was associated with conjunctival hyperaemia. Ophthalmologist evaluation assessed the absence of vision impairment and corneal involvement and a topical steroid and antibiotic therapy was started along with right ocular bandage. On the fifth day of hospitalization, tecovirimat became available into our centre and treatment was promptly started at the standard dose of 600 mg, twice daily for 14 days. The treatment was well-tolerated without any adverse events or significant blood test alterations. After starting tecovirimat, a rapid clinical improvement was observed with the asynchronous evolution of all the cutaneous lesions and the absence of new lesions after 48 h from treatment initiation and the rapid resolution of mucosal involvement.

**Figure 1:**
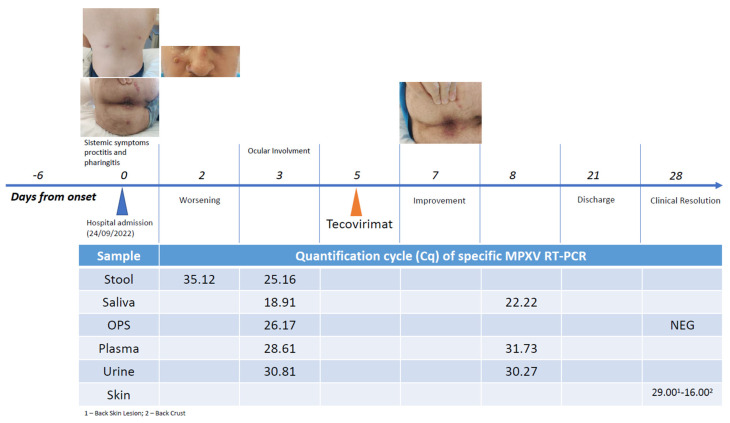
Timeline of clinical evolution and PCR positivity in biological samples collected.

During the hospitalization, biological samples from multiple sites (OPS, urine, saliva, plasma and stool) were collected for virological investigation. Viral DNA was extracted by the automatic extractor QIAsymphony (Qiagen, Hilden, Germany) and amplified using the real-time PCR method published by Li et al. [16], targeting the TNF (tumour necrosis factor) receptor gene, G2R. MPXV DNA concentration was measured using threshold cycles (Ct) values of the MPXV-specific PCR. Samples with Ct values higher than 40 were considered negative. The viral kinetics from multiple sites and the patient’s clinical evolution at different time points are shown in Figure 1.

The patient was discharged in good clinical conditions with complete resolution of the proctitis and pharyngotonsillitis, preserved vision and residual crusted lesions on the feet and on his back. He self-isolated at home until complete clinical resolution which occurred 38 days after symptom onset.

## 3. Discussion

We described, for the first time to the best of our knowledge, a case of invasive bacterial disease due to *S. pyogenes* in a HIV-positive patient with Mpox and extensive muco-cutaneous involvement.

Secondary bacterial infections have been frequently reported in subjects with Mpox [13,17], mostly as mild and localized, usually related to superinfection of the muco-cutaneous lesions [17]. A previous prospective cohort study about the management of bacterial infections in Mpox outpatients showed that 15 of 129 (11.6%) patients developed bacterial superinfections, of whom, only 2.3% requiring hospitalization [15]. In addition, another retrospective cohort study from the United Kingdom that analysed 142 hospitalized Mpox patients demonstrated that secondary bacterial infections are a frequent complication (58%) of the disease with the most common manifestations being cellulitis and pharyngitis and only one case of *Escherichia coli* bacteraemia [13]. On the contrary, secondary bacterial infections causing generalized infections or sepsis have rarely been reported [13], especially in subjects with underlying comorbidities. A recent study showed that 76 (20%) of 382 advanced HIV individuals with Mpox reported secondary bacterial infections, including cellulitis, abscesses and sepsis (4.4%). Among these latter, only eight had positive blood cultures due to Gram-negative bacteria and polymicrobial agents. Superinfections were more common in Mpox patients with advanced HIV infection (CD4 count < 100 cells/mmc), suggesting that the incidence of bacterial infection was inversely proportional to CD4 count [14].

*S. pyogenes* (*group A Streptococcus*, GAS) is a facultative anaerobic Gram-positive coccus, mainly associated with pharyngitis and skin/soft-tissue infections (SSTIs) and more rarely responsible for invasive disease, which includes necrotizing skin infections, bacteraemia, pregnancy-associated infections and respiratory tract infections [18,19,20]. The skin and the upper respiratory tract are major reservoirs for GAS infection. Infections caused by GAS at these anatomical sites give rise to two physiological processes: bacterial adhesion and colonization. For these specific reasons, GAS may cause skin colonization, leading to superficial infections such as pyoderma [21].Moreover, from the skin, GAS can invade deeper tissues, leading to GAS invasive disease [22].

The most common portals of entry for GAS invasive disease involve skin, oral and vaginal mucosa [23], especially during SSTIs when a loss of the integrity of the muco-cutaneous barrier occurs [24]. In our patient, multiple skin lesions were swabbed and tested positive for Mpox. Unfortunately, skin lesions were not tested for *S. pyogenes*. Indeed, blood cultures carried out upon patient arrival were positive for *S. pyogenes*. For these reasons, we can assume that *S. pyogenes* may have colonized the skin and the oral mucosa where lesions and inflammation probably facilitated the translocation of *S. pyogenes* into the bloodstream, leading to an invasive GAS infection.

Furthermore, our patient was affected by HIV and this may have played a role in the acquisition of GAS bacteraemia. In fact, it has been shown that HIV infection is an independent risk factor for GAS invasive disease [25]. Of note, in our case, although initial clinical presentation could be entirely due to Mpox, the prompt execution of blood cultures at hospital admission allowed the bacteraemia to be identified and targeted antimicrobial therapy to be quickly set up.

Regarding Mpox clinical presentation, our clinical case was characterized by a wide extension of muco-cutaneous lesions and multi-mucosal involvement with pharyngotonsillitis, severe proctitis and ocular lesions. HIV co-infection, although immunologically and virologically well controlled, might have contributed to the progression to severe disease. The association between HIV-related advanced immunosuppression and a more severe course of Mpox disease has been clearly demonstrated both in previous outbreaks [26] and in the 2022 global outbreak. Specifically, a recent multicentric study showed that HIV-positive patients with advanced immunosuppression (CD4 count < 200 cell/mm^3^) more frequently experienced severe evolution protracted illness with fulminant disseminated necrotising cutaneous lesions, systemic complications, and a high mortality rate (25%) [14]. Moreover, in these patients, bacterial secondary infections leading to septic shock and multi-organ failure were the most frequent causes of death (20/27 subjects) [14]. On the contrary, the evolution of Mpox in people living with HIV (PLWH) with a good viroimmunological status is more controversial. In fact, although previous data reported similar clinical outcomes in subjects with well-controlled HIV infections compared with subjects without HIV [27,28,29], a more protracted course of the disease [30] with a higher frequency of symptomatic diseases, particularly rash and anorectal symptoms/proctitis [31,32] and a greater utilization of healthcare resources and rates of hospitalizations [31,32] have been described in PLWH compared to their counterparts. Furthermore, an exuberant cutaneous presentation has been described in an acute HIV setting [33,34]. These data suggest that an early start of Mpox antiviral treatments and pre-exposure prophylaxis in PLWH, regardless of immune status, might be important to prevent the progression to severe disease and complications [30]. Tecovirimat is an antiviral drug approved for smallpox treatment and currently authorized for Mpox [35,36] based on efficacy data in animal studies [37,38]. Although data from case series reported good tolerability and safety [39,40], with similar outcomes in HIV-infected and non-infected patients [41], contrasting and limited evidence on its in vivoefficacy are still available [42]. In our patient, tecovirimat treatment was started on the basis of clinical severity and, despite the delay in treatment initiation due to the lack of drug availability, it was effective, with a rapid clinical improvement and the absence of new lesions after 48 h of treatment.

Finally, in our patient, viral DNA was detected in the stool, skin lesions, saliva, urine, OPS and plasma. Although follow-up biological samples were not all collected at the same time points, skin lesions seem to be the samples with the higher viral loads and the longest clearance time. This finding is in line with recent studies showing that cutaneous lesions were the samples with the longest median time to viral clearance (25 days), followed by oropharyngeal and rectal samples [43], and with the highest viral load [44].

A recent systematic review and meta-analysis estimated the skin viral burden in Mpox patients including 731 out of 790 confirmed Mpox individuals demonstrating that the pooled skin viral load was 21.71 (95% CI: 20.68–22.75) [45]. This study postulated that the skin is a viral reservoir and that the elevated Mpox viral loads (lower Ct values: a reverse correlation exists between Ct values and viral loads [46]) in association with a high rate of cutaneous lesions positive for Mpox indicate a potential transmission route via direct contact with skin lesions [45]. Our Spanish colleagues demonstrated that specimens from the oropharynx and rectum contained replication-competent viruses, showing other possible sources of infection; however, the viral DNA loads were lower, suggesting that replication-competent viruses were less common in these samples in comparison to those taken from the skin [44]. Furthermore, our French colleagues demonstrated that MPXV viral loads were high in the skin and the anus, intermediate in the throat and semen and low in the urine and the blood, confirming a possible sexual transmission [47]. According to this result, a recent study comparing viral MPXV DNA detection and replication-competent viruses from viral cultures showed that specimens with a higher viral burden are more likely to result in positive cultures and subsequently, in heightened viral infectivity. In particular, high viral loads and positive cultures were observed in skin and anal lesions [48]. For these reasons, viral load monitoring can be useful in creating a better understand of the possible routes of MPVX transmission and to evaluate the correlation between PCR positivity and associated infectivity according to the lesions’ anatomical locations.

In addition to this, studies that investigated viral loads in skin samples from individuals with Mpox have revealed that children [49,50] and generally patients with severe Mpox, tend to have higher viral load compared to Mpox adult individuals without severe disease [49,50]. In particular, one study revealed that paediatric patients had a higher likelihood of requiring admission to the Intensive Care Unit compared to adults [49]. For these reasons it may be possible to use viral loads in skin samples as a biomarker forMpox severity, or as a predictor for Mpox disease progression and prognosis [45].

Most of these studies included male Mpox cases of all ages from non-endemic countries. Certainly, larger studies including patients with different characteristics and originating from both endemic and non-endemic countries would be useful in determining the role of Mpox viral load in predicting severity, infectivity and the prognosis of a specific Mpox infection. Moreover, further studies, ideally involving analysis of viral cultures, are needed in order to better comprehend viral infectivity.

## 4. Conclusions

This is a case of Mpox with multiple complications such as ocular, pharyngeal and rectal involvement, wide cutaneous extension of the rash and concurrent *S. pyogenes* bacteraemia which was probably related to bacterial translocation from the skin or oral cavity. This widespread muco-cutaneous extension of the Mpox lesions poses a risk of secondary bacterial infections due to compromised skin integrity. Bacterial superinfections such as SSTIs in Mpox can be challenging to diagnose due to the overlap between clinical manifestations. Furthermore, localized bacterial infections can potentially progress to invasive forms, such as sepsis. For these reasons, bacteriological investigations in Mpox patients are essential to set up the correct antimicrobial therapy, when necessary.

Notably, in our patient, HIV infection, despite being well controlled through antiretroviral therapy, may have contributed to the severe course of MPXV infection. HIV weakens the immune system, leaving individuals more vulnerable to infections and increasing the likelihood of complications. Therefore, prompt initiation of antiviral treatment is crucial, particularly in HIV-positive patients, to reduce the onset and severity of complications.

## Data Availability

No new data were created or analysed in this study. Data sharing is no applicable to this article.

## Abbreviations

Monkeypox virus (MPXV), men who have sex with men (MSM), antiretroviral therapy (ART), sexually transmitted diseases (STDs), oropharyngeal swab (OPS), group A Streptococcus (GAS), people living with HIV infection (PLWH), reverse transcriptase polymerase chain reaction (RT-PCR), tumour necrosis factor (TNF), threshold cycles (Ct), skin/soft-tissue infections (SSTIs).
